# Supplementary material for: Flow-driven construction of capillary-scale vessels with predefined geometries in natural hydrogels
Source: Mater Today Bio. 2025 Oct 18;35:102433. doi: 10.1016/j.mtbio.2025.102433 (PMC12630036; doi:10.1016/j.mtbio.2025.102433)
Supplement: Multimedia component 1 [file mmc1.docx]

**Supplementary Fig. 1 Hollow structures fabricated along single-layer zigzag trajectory at various reciprocating intervals.** (A, B) Confocal reflection images of the hollow structures in collagen gel fabricated along the single-layer trajectory at irradiation intensities of 20 and 15 mW. Scale bars, 20 μm. (C, D) Quantification of the width of the hollow structures in collagen gel. (E, F) Confocal reflection images of the hollow structures in fibrin-collagen gel fabricated along the single-layer trajectory at irradiation intensities of 20 and 15 mW. Scale bars, 20 μm. (G, H) Quantification of the width of the hollow structures in fibrin-collagen gel. Data are shown as the mean ± SD. n=3.
