# Supplementary material for: Flow-driven construction of capillary-scale vessels with predefined geometries in natural hydrogels
Source: Mater Today Bio. 2025 Oct 18;35:102433. doi: 10.1016/j.mtbio.2025.102433 (PMC12630036; doi:10.1016/j.mtbio.2025.102433)
Supplement: Multimedia component 2 [file mmc2.docx]

**Supplementary Fig. 2 Hollow structures fabricated along single-layer straight-line trajectory at various frequencies.** (A, B) Confocal reflection images of the hollow structures in collagen gel and fibrin-collagen gel fabricated along the single-layer straight-line trajectory. Scale bars, 20 μm. (C, D) Quantification of the width of the hollow structures in collagen gel and fibrin-collagen gel. Data are shown as the mean ± SD. n=51.
