# Supplementary material for: Flow-driven construction of capillary-scale vessels with predefined geometries in natural hydrogels
Source: Mater Today Bio. 2025 Oct 18;35:102433. doi: 10.1016/j.mtbio.2025.102433 (PMC12630036; doi:10.1016/j.mtbio.2025.102433)
Supplement: Multimedia component 3 [file mmc3.docx]

**Supplementary Fig. 3 Hollow structures fabricated along single-layer straight-line trajectory at various stage speed.** (A) Confocal reflection images of the hollow structures in collagen gel fabricated along the single-layer straight-line trajectory. Scale bars, 10 μm. (B) Confocal reflection images of the hollow structures in fibrin-collagen gel fabricated along the single-layer straight-line trajectory. Scale bars, 10 μm.
