# Supplementary material for: Flow-driven construction of capillary-scale vessels with predefined geometries in natural hydrogels
Source: Mater Today Bio. 2025 Oct 18;35:102433. doi: 10.1016/j.mtbio.2025.102433 (PMC12630036; doi:10.1016/j.mtbio.2025.102433)
Supplement: Multimedia component 4 [file mmc4.docx]

**Supplementary Fig. 4 Hollow structures fabricated along five-layer zigzag trajectory at various irradiation intervals.** (A) Schematic illustration of the five-layer zigzag trajectory. (B, C) Confocal reflection images of the hollow structures in collagen gel and fibrin-collagen gel fabricated along the five-layer zigzag trajectory. Scale bars, 20 μm. (D, E) Quantification of the width of the hollow structures in collagen gel and fibrin-collagen gel. Data are shown as the mean ± SD. n=51.
