# Supplementary material for: Flow-driven construction of capillary-scale vessels with predefined geometries in natural hydrogels
Source: Mater Today Bio. 2025 Oct 18;35:102433. doi: 10.1016/j.mtbio.2025.102433 (PMC12630036; doi:10.1016/j.mtbio.2025.102433)
Supplement: Multimedia component 5 [file mmc5.docx]

**Supplementary Fig. 5 Perfusability and endothelial barrier function of microvessels formed along 20 µm microchannels.** (A) Phase-contrast image of a microvessel formed under flow conditions. Scale bar, 50 μm. (B) Confocal fluorescence images showing perfusion of 70 kDa FITC-dextran through the microvessel. Scale bar, 100 μm.
