# Supplementary material for: Flow-driven construction of capillary-scale vessels with predefined geometries in natural hydrogels
Source: Mater Today Bio. 2025 Oct 18;35:102433. doi: 10.1016/j.mtbio.2025.102433 (PMC12630036; doi:10.1016/j.mtbio.2025.102433)
Supplement: Multimedia component 6 [file mmc6.docx]

**Supplementary Fig. 6 Vascular formation along a 20 μm diameter channel in collagen gel.** (A) Phase-contrast images of vascular formation under static and flow condition (0, 5, 10 mm H_2_O). Scale bars, 100 μm. (B) Immunofluorescence images of vascular formation under static and flow condition (0, 5, 10 mm H_2_O). Cells were fixed on day 7 and stained for nuclei (Hoechst 33342) and F-actin (phalloidin). Scale bars, 100 μm.
