# Supplementary material for: Flow-driven construction of capillary-scale vessels with predefined geometries in natural hydrogels
Source: Mater Today Bio. 2025 Oct 18;35:102433. doi: 10.1016/j.mtbio.2025.102433 (PMC12630036; doi:10.1016/j.mtbio.2025.102433)
Supplement: Multimedia component 8 [file mmc8.docx]

**Supplementary Fig. 8 Expression of vimentin during vascular formation in a 20 μm diameter channel.** (A, B) Phase-contrast images of HUVECs on day 3 under static and flow conditions. (C–H) Fluorescence projection images of cells within straight microchannels in the root, middle, and tip regions, which are corresponding to the cross-sectional images in Fig. 6. Cells were fixed on day 3 and stained for vimentin (red), and nuclei (Hoechst 33342, blue). Scale bar, 30 μm.
