# Supplementary material for: Flow-driven construction of capillary-scale vessels with predefined geometries in natural hydrogels
Source: Mater Today Bio. 2025 Oct 18;35:102433. doi: 10.1016/j.mtbio.2025.102433 (PMC12630036; doi:10.1016/j.mtbio.2025.102433)
Supplement: Multimedia component 9 [file mmc9.docx]

**Supplementary Movie 1 Time-lapse movie of the cells under static conditions.** Sequential phase-contrast images were obtained by phase-contrast microscope equipped with a time-lapse imaging system. Images were obtained at 15 min intervals for 24 h starting on day 2.

**Supplementary Movie 2 Time-lapse movie of the cells under flow conditions (10 mmH_2_O).** Sequential phase-contrast images were obtained by phase-contrast microscope equipped with a time-lapse imaging system. Images were obtained at 15 min intervals for 24 h starting on day 2.

**Supplementary Movie 3 Image correlation analysis movie under static conditions.** Supplementary movie 1 was analyzed for visualizing the movement of each cell.

**Supplementary Movie 4 Image correlation analysis movie under flow conditions.** Supplementary movie 2 was analyzed for visualizing the movement of each cell.
